# Structural characterization of aspartate-semialdehyde dehydrogenase from *Pseudomonas aeruginosa* and *Neisseria gonorrhoeae*

**DOI:** 10.1038/s41598-022-17384-9

**Published:** 2022-08-17

**Authors:** S. L. Teakel, J. W. Fairman, M. M. Muruthi, J. Abendroth, D. M. Dranow, D. D. Lorimer, P. J. Myler, T. E. Edwards, J. K. Forwood

**Affiliations:** 1 School of Dentistry and Medical Sciences, Charles Sturt University, Wagga Wagga, NSW 2650 Australia; 2 Genentech, 1 DNA Way, South San Francisco, CA 94080 USA; 3 Seattle Structural Genomics Center for Infectious Disease (SSGCID), Seattle, WA 98109 USA; 4 Beryllium Discovery Corp, 7869 NE Day Road West, Bainbridge Island, WA 98110 USA; 5 UCB, Bainbridge Island, WA 98110 USA; 6 Seattle Children’s Research Institute, University of Washington, Seattle, WA USA

**Keywords:** Oxidoreductases, X-ray crystallography

## Abstract

Gonorrhoea infection rates and the risk of infection from opportunistic pathogens including *P. aeruginosa* have both risen globally, in part due to increasing broad-spectrum antibiotic resistance. Development of new antimicrobial drugs is necessary and urgent to counter infections from drug resistant bacteria. Aspartate-semialdehyde dehydrogenase (ASADH) is a key enzyme in the aspartate biosynthetic pathway, which is critical for amino acid and metabolite biosynthesis in most microorganisms including important human pathogens. Here we present the first structures of two ASADH proteins from *N. gonorrhoeae* and *P. aeruginosa* solved by X-ray crystallography. These high-resolution structures present an ideal platform for in silico drug design, offering potential targets for antimicrobial drug development as emerging multidrug resistant strains of bacteria become more prevalent.

## Introduction

**Figure 1:**
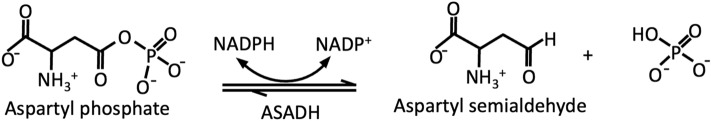
Conversion of aspartyl phosphate to aspartyl semialdehyde catalyzed by ASADH.

Aspartate-semialdehyde dehydrogenase (ASADH; EC 1.2.1.11) is an enzyme involved in the aspartate biosynthetic pathway, responsible for catalyzing the reductive dephosphorylation of aspartyl phosphate into aspartate semialdehyde (Fig. 1)^1^. The pathway is required for production of essential amino acids and metabolites in microorganisms and has proved an attractive target for antimicrobial drugs^2–7^.

An emerging challenge for human health is the increasing microbial resistance against several of the most commonly used antibiotics. *Neisseria gonorrhoeae* and *Pseudomonas aeruginosa* are both gram-negative pathogenic bacteria. *P. aeruginosa* is a gram-negative bacterium and an opportunistic pathogen in humans and plants. In healthy individuals it is responsible for mild or asymptomatic infections, however immunocompromised individuals, patients with cystic fibrosis, newborns and pre-term, or low birth weight neonates are at higher risk of acute *P. aeruginosa* infection and/or complications^8,9^. *P. aeruginosa* displays an adaptive resistance that involves biofilm formation, which can act as a diffusion barrier to antibiotics in the lungs^8^, and antibiotic resistance is an increasing challenge in the treatment of infection^10^*. P. aeruginosa* exhibits antibiotic resistance through a variety of mechanisms including horizontal transfer of resistance genes and/or gene mutations, expression of efflux pumps that remove antibiotics from the cell, production of enzymes that inactivate the antibiotic, and a low outer membrane permeability^8^. *Pseudomonas mallei* and *Pseudomonas pseudomallei* (also known as *Burkholderia mallei* and *Burkholderia pseudomallei*) are two pathogenic organisms closely related to *P. aeruginosa* that are also responsible for the human diseases, including glanders and melioidosis, respectively^11^.

*Neisseria meningitides and N. gonorrhoeae* are two pathogenic species responsible for diseases such as gonorrhoea, cervicitis, urethritis, pharyngitis, proctitis and occasionally conjunctivitis. Newborn exposure to an *N. gonorrhoeae* infection during birth can result in ocular infections (ophthalmia neonatorum). Common antimicrobial drugs used to treat a variety of infectious microorganisms, including doxycycline and erythromycin, increase selective pressure on infectious agents such as *N. gonorrhoeae*^12^ and these selective pressures enhance the emergence of resistant strains of *N. gonorrhoeae*^13–17^. Of further concern, *N. gonorrhoeae* is a cofactor that can facilitate HIV-1 infection^18^. In 2015, in response to the threat of antimicrobial resistance the World Health Organization (WHO) adopted a global action plan. The plan briefly involves increased awareness and understanding, increased surveillance and research, increased preventative measures, increased investment for treatment, diagnosis, vaccination and other interventions, and to ensure that the plan is inclusive for all countries^19^. The design of new antimicrobial drugs is critical to counter emerging multidrug-resistant pathogenic microorganisms.

ASADH has been identified as a target for antimicrobial drugs in both pathogenic and non-pathogenic microorganisms including gram-negative bacteria^20,21^, gram-positive bacteria^22^, *Mycobacterium tuberculosis*^23^, and archael^24^ and fungal species^2,25,26^. Humans and other mammals do not have a homolog of ASADH or require the aspartate biosynthetic pathway, making this a highly attractive pathway to target in microorganisms. Some organisms possess a second, redundant ASADH enzyme, which despite sharing a high structural similarity has less than 50% sequence identity to other functional ASADH proteins^27^. Despite ASADH sharing an overall high structural similarity between fungal and bacterial orthologs, secondary structural differences have been identified that offer the potential for the development of species-selective antimicrobial drugs^2^.

Several antibiotic resistance genes have been identified within the *P. aeruginosa* genome and antibiotic resistance through horizontal gene transfer has been demonstrated^8,28^. Therefore, new inhibitors are required to combat antibiotic resistance. For microorganisms that use the aspartate biosynthesis pathway for the production of essential amino acids and metabolites, inhibition of ASADH is fatal^29^. The *asd* gene encoding ASADH is one of the minimal genes essential for life in these microorganisms^7,30–33^.

Currently, inhibitors are used in combination with various antibiotics on antimicrobial-resistant pathogens^34^ and the use of antibiotic adjuvants can effectively reduce bacterial resistance to commonly used antibiotics^35,36^*.* Selective inhibition of key enzymes for biosynthesis pathways in microorganisms can be used to counter antimicrobial drug resistance of pathogenic species such as *P. aeruginosa.* To provide an enhanced understanding of the structural similarities of ASADH between important microorganisms, as well as to provide a platform for the design of new anti-microbials, here we solve and analyze the structures of two ASADH enzymes from *N. gonorrhoeae* and *P. aeruginosa.*

## Materials and methods

ASADH proteins were purified using standardized protein expression and purification protocols as described previously^37^. Briefly, open reading frames encoding ASADH were amplified from genomic DNA and cloned into an expression vector encoding an N-terminal non-cleavable 6-His tag. The proteins were expressed in BL21(DE3) bacterial cells and then purified by nickel affinity and size-exclusion chromatography^37^. The proteins were concentrated to ~ 20 mg/mL and stored at − 80 °C until used in crystallography experiments. The sparse matrix screening method was used to produce crystals from purified ASADH proteins via the sitting drop vapor diffusion method. Crystals of *P. aeruginosa* ASADH (SSGCID ID: PsaeA.17885.a.B1.PS02341) were grown at 19.6 mg/ml with 4 mM NADP, and mixed 1:1 with Microlytic MCSG1 screen, well D9 (containing 0.2 M sodium chloride, 0.1 M TRIS:HCl pH 8.50, 25% PEG3350) at 289 K. Crystals harvested were cryo-protected with crystallant supplemented with 15% ethylene glycol and 5 mM NADP and flash‐cooled in liquid nitrogen. X-ray diffraction data with a resolution of 2.1 Å was collected at a temperature of 100 K on a RAYONIX MX-225 CCD detector at a wavelength of 0.9787 Å on the Advanced Photon Source (APS, Argonne, IL) 21‐ID‐F beamline. Crystals from *N. gonorrhoeae* ASADH (SSGCID ID: NegoA.17885.a.B1.PW37925) were obtained in a condition from Microlytic MCSG1 screen (F11) containing 25% PEG 3350, 200 mM ammonium sulphate, HEPES free acid/NaOH buffer pH 7.5 at temperature 290 K. X-ray diffraction data with a resolution of 2.1 Å was collected at a temperature of 100 K on the RIGAKU SATURN 944 + CCD detector at a wavelength of 1.5418 Å. The diffraction data were reduced using the XDS/XSCALE package^38,39^. The structures of the *P. aeruginosa* and *N. gonorrhoeae* ASADH proteins were solved by molecular replacement using ASADH proteins as starting models from *Vibrio cholerae* (1MB4) in BALBES^40^ and *Burkholderia thailandensis* (3UW3) in MoRDa respectively. Protein structures were refined using PHENIX^41^. Protein structures shown were modelled using PyMOL and SeeSAR version 9.2 (BioSolveIT GmbH) software^42^. SeeSAR was utilised to model docking of the SMCS molecule with ASADH proteins. SeeSAR calculates estimated affinities and allows the selection of best poses based on HYDE scoring and analysis of torsion. The default parameter settings were used. The model adopted a similar binding site for docking as defined by “find unoccupied pocket”. From the molecular docking, parameters such as estimated affinity and torsion were acknowledged in selecting the best model. DALI^43^ (heuristic PDB search) protein structure comparison by alignment of distance matrices were performed. The final protein structures were deposited into the Protein Data Bank (PDB) and assigned the codes 6BAC for *N. gonorrhoeae* ASADH and 5BNT for *P. aeruginosa* ASADH.

## Results and discussion

### Structural analysis of ASADH proteins from *N. gonorrhoeae *and *P. aeruginosa*

The structures of ASADH proteins from *N. gonorrhoeae* and *P. aeruginosa* were determined using x-ray crystallography, deposited into the Protein Data Bank and assigned the PDB codes 6BAC and 5BNT, respectively (Table 1). Crystals of both ASADH proteins produced x-ray diffraction data with a resolution of 2.1 Å.

**Table 1:** Crystallography data collection and refinement statistics.

	5BNT	6BAC
Resolution range (Å)	43.484–2.10	39.54–2.10
Space group	P 41 21 2	P 31 2 1
Unit cell length (Å)	a = 125.68 b = 125.68 c = 199.44	a = 109.00 b = 109.00 c = 72.40 Å
Unique reflections	90,696	29,204
R-pim	0.133	0.077
Completeness (%)	96.9	99.5
Mean I/Sigma (I)	15.13 (3.59)	13.44 (2.98)
Total number of atoms	12,304	3038
RMS (bonds) (Å)	0.83	0.37
RMS (angles) (°)	1.10	0.54
Wilson B Factor	19.55	39.2
R-merge	0.125	0.07
R-work	0.182	0.180
R-free	0.220	0.228
Protein Atoms	11,169	2767
Solvent Atoms	928	222
Heterogen Atoms	157	41
Average B-factor (Å2 )	24.0	51.0
Clashscore	2	2.10
Ramachandran favoured (%)	96	97
Ramachandran allowed (%)	4	3
Ramachandran outliers (%)	0	0
Rotamer Outliers (%)	0	1

**Figure 2:**
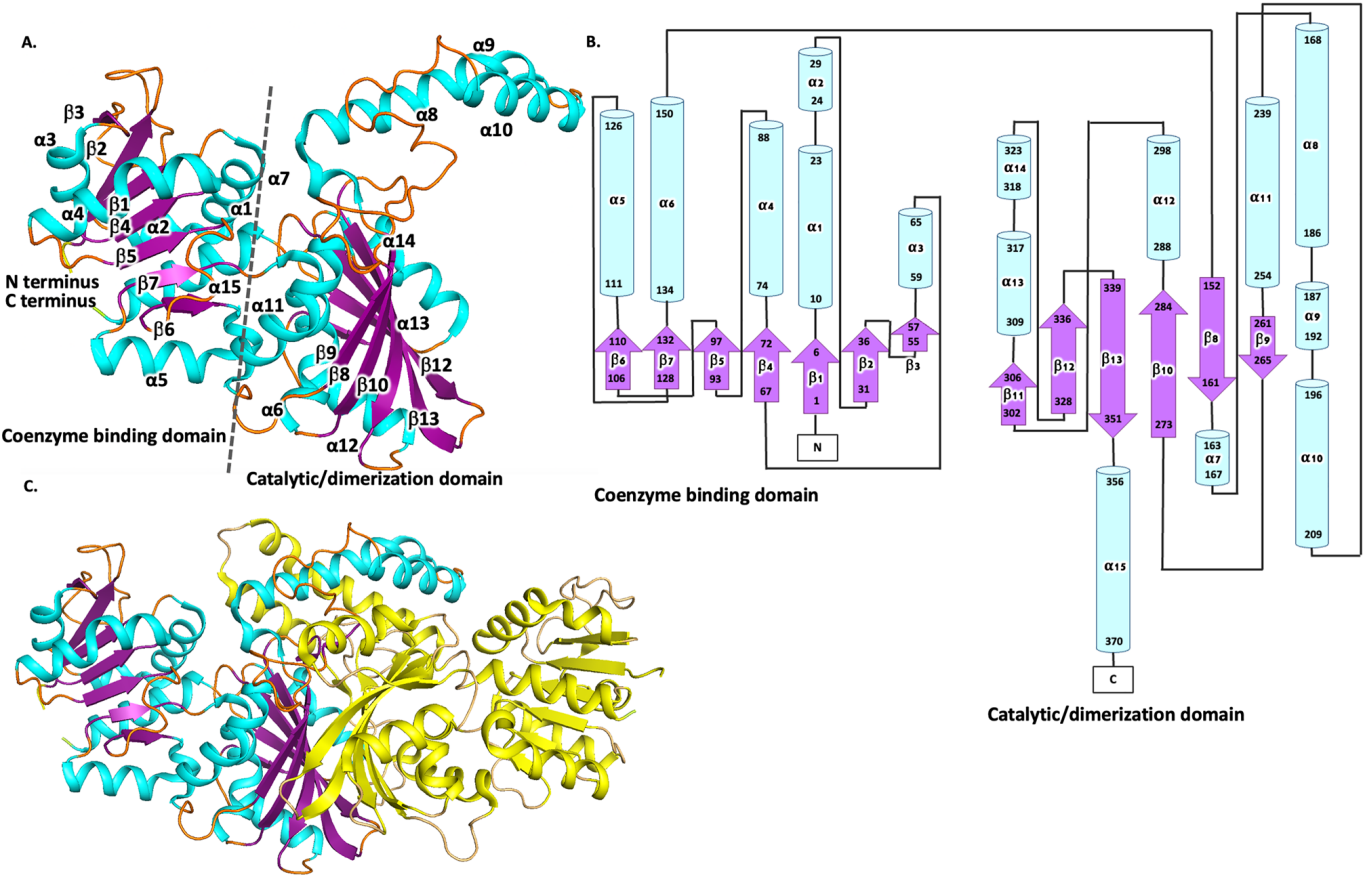
Schematic representation of the structure of ASADH from *N. gonorrhoeae* (6BAC)*.* (**A**) Monomeric *N. gonorrhoeae* ASADH, with α helices colored cyan and β-sheets colored purple. The N- and C-termini are shown in yellow and green, respectively. (**B**) Topology map of *N. gonorrhoeae* ASADH showing helices (cylindrical) and directional beta sheets (arrows) analyzed in PDBsum^47^. (**C**) *N. gonorrhoeae* ASADH dimer, with one monomer colored as for A, and the second monomer colored in yellow.

The crystallographic structure from *N. gonorrhoeae* ASADH was solved in the space group P 31 2 1, with unit cell dimensions of *a* = 109 Å, *b* = 109 Å, and *c* = 72.4 Å, and one ASADH protein was present in the asymmetric unit. Continuous electron density allowed modelling of all 371 residues and included two His residues of the non-cleavable N-terminal polyhistidine tag (MAHHHHHH) present in the structure at the N terminus of the protein. The enzyme structure adopted a well-described coenzyme binding domain at the N-terminus (residues 1–150) comprised of six α-helices and seven β-strands, and a catalytic and dimerization domain at the C-terminus (residues 152–351; Fig. 2A,B). The parallel β-sheet flanked by two alpha-helical bundles forms a Rossmann fold, which typically binds enzyme cofactors including β-nicotinamide adenine dinucleotide (NAD) and β-nicotinamide adenine dinucleotide phosphate (NADP)^44^. The highest structural homology to *N. gonorrhoeae* ASADH*,* was the ASADH enzyme from *B. thailandensis* (3UW3)^45^, with an RMSD of 0.7 Å (Table 2).

**Table 2:** Comparison of structural homology of ASADH proteins to *P. aeruginosa* ASADH (5BNT) and *N. gonorrhoeae* ASADH (6BAC).

PDB ID	z		RMSD		LALI		%ID		Nres	Organism
	5BNT	6BAC	5BNT	6BAC	5BNT	6BAC	5BNT	6BAC		
6BAC	55.9	–	0.8	–	371	–	68	–	371	*N. gonorrhoeae*
3PZR	57.2	55.7	0.5	0.8	369	370	69	66	371	*V. cholerae*
3UW3	56.1	56.1	0.8	0.7	370	370	73	71	375	*B. thailandensis*
1PQU	56.0	54.5	0.6	0.9	369	369	67	66	372	*H. influenzae*
1GL3	53.5	50.7	1.1	0.8	367	355	69	66	368	*E. coli*
4WOJ	52.2	51.0	1.3	1.5	363	362	51	46	365	*F. tularensis subsp. Tularensis*
2GYY	39.9	39.3	2.0	2.1	332	330	23	23	352	*S. pneumoniae*
3TZ6	39.2	38.3	2.2	2.4	335	335	25	24	343	*M. tuberculosis H37Rv*
2YV3	38.7	37.6	2.2	2.3	325	322	29	27	328	*T. thermophilus HB8*
1YS4	31.9	30.8	2.8	2.9	304	302	20	22	348	*M. jannaschii*
4ZIC	31.6	30.7	2.6	2.8	303	302	19	21	357	*T. rubrum*
5JW6	31.3	30.9	2.7	2.9	302	304	21	20	358	*A. fumigatus*
6C85	31.0	30.5	2.7	2.8	303	302	18	20	354	*B. dermatitidis*
5CEF	29.9	28.8	2.5	2.7	301	300	20	21	359	*C. neoformans*
3HSK	29.4	28.7	2.8	2.8	299	358	20	19	358	*C. albicans*

*Z* z-score confidence in similarity significance, *RMSD* Root mean square deviation across the aligned sequences, *LALI* Total number of aligned residues, *Nres* Total number of residues, *%ID* Percent identity. PDB structures listed below the line were limited to 1 structure per species.

To determine whether *N. gonorrhoeae* ASADH was likely to form a multimer, we examined the crystal structure by expanding the asymmetric unit. The biological assembly for the ASADH enzyme (6BAC), predicted using Proteins, Interfaces, Structures and Assemblies (PISA), was a dimer with a large interface present between two protomers^46^ (Fig. 2C). Overall, the protein interface exhibits 52 hydrogen bonds, 18 salt bridges and 3406 Å^2^ of buried surface area. This biological interface was structurally equivalent to the ASADH protein dimer from *B. thailandensis* (PDB 3UW3) which exhibited 47 hydrogen bonds, 17 salt bridges and 3463 Å^2^ of buried surface area.

**Figure 3:**
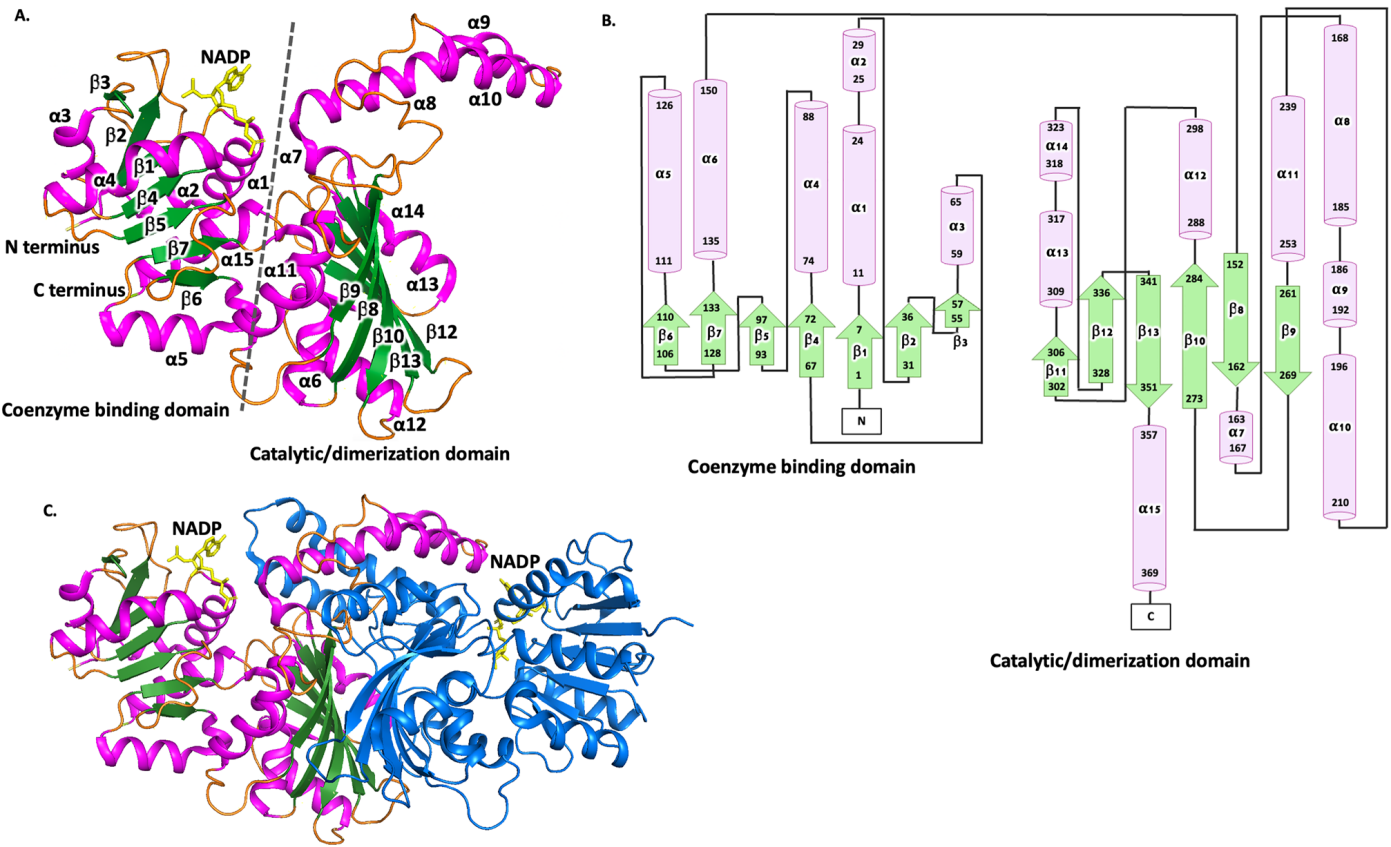
Schematic representation of the structure of ASADH from *P. aeruginosa* (5BNT)*.* (**A**) *P. aeruginosa* ASADH structure with α helices colored pink and β-sheets colored green. The N terminus (pale yellow) and C terminus (pale green) are depicted. (**B**) Topology map of *P. aeruginosa* ASADH showing helices (cylindrical) and directional beta sheets (arrows) obtained from PDBsum^47^. (**C**) *P. aeruginosa* ASADH formed a dimer with one NADP molecule (yellow) bound within each protomer.

The structure of the *P. aeruginosa* ASADH was solved in the space group P 41 21 2, with unit cell dimensions of *a* = 125.68, *b* = 125.68, and *c* = 199.44. The asymmetric unit contained four ASADH protomers arranged as two dimers. The electron density allowed for modelling of residues 1–370. Each monomer is comprised of an N-terminal coenzyme-binding domain (residues 1–133) and a C-terminal catalytic and dimerization domain (residues 135–351), with similar architecture and topology to the ASADH from *N. gonorrhoeae* (Fig. 3A,B). PISA analysis identified a dimer as the biological unit (Fig. 3C), with the protein interface comprised of 56 hydrogen bonds, 23 salt bridges and 3558 Å^2^ of buried surface area^46^. This interface was comparable to ASADH enzymes exhibiting NADP binding including *Haemophilus influenzae* ASADH (1PQU) involving 52 hydrogen bonds, 18 salt bridges and 3415 Å^2^ of buried surface area, and *V. cholerae* ASADH (3PZR) involving 60 hydrogen bonds, 24 salt bridges and 3451 Å^2^ of buried surface area. In addition, there was a high structural similarity between *P. aeruginosa* ASADH (5BNT) and the *Haemophilus influenzae* (1PQU) and *V. cholerae* (3PZR) ASADH proteins, with RMSD of 0.6 Å and 0.5 Å respectively as determined in DALI^48^.

### NADP binding in *P. aeruginosa* ASADH

**Figure 4:**
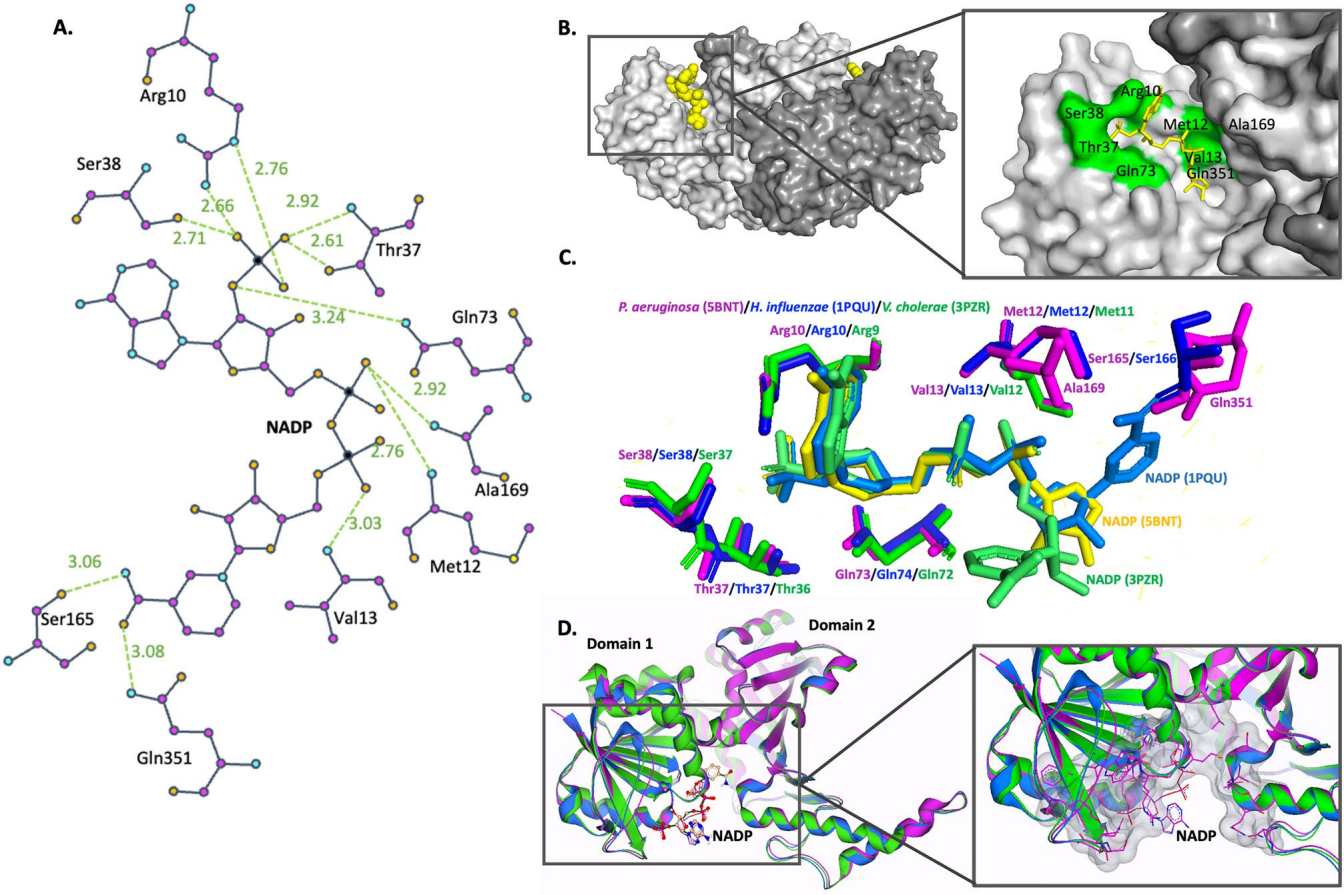
NADP binding in *P. aeruginosa* (5BNT), *H. influenzae* (1PQU) and *V. cholerae* (3PZR) ASADH proteins. (**A**) Schematic of hydrogen bonds (H-bond) between bonding residues of *P. aeruginosa* ASADH and NADP. (**B**) NADP (yellow) bound within the *P. aeruginosa* ASADH dimer and active site residues within the NADP binding site cavity that are important for H-bonding (green). (**C**) Residues responsible for NADP H-bonding in *P. aeruginosa* (5BNT, purple), *H. influenzae* (1PQU, blue) and *V. cholerae* (3PZR, green) show several conserved residues in NADP binding. (**D**) Superimposed structures of *P. aeruginosa* (5BNT, purple), *H. influenzae* (1PQU, blue) and *V. cholerae* (3PZR, green) showing the binding surface of the ASADH proteins (grey) modelled in SeeSAR Version 9.2^42^.

*P. aeruginosa* ASADH was co-crystallized with NADP. Each monomer of *P. aeruginosa* ASADH bound a single NADP molecule (Fig. 4A,B). To examine the conservation of NADP binding residues, structurally similar ASADH proteins exhibiting NADP binding were compared (Fig. 4C). We found many NADP binding residues to be strictly conserved between *P. aeruginosa* (5BNT) and two structurally similar ASADH proteins; *H. influenzae* (1PQU) and *V. cholerae* (3PZR) (Fig. 4D). Overall, the *P. aeruginosa* ASADH-NADP interface was mediated through 14 hydrogen bonds with a buried surface area of 582.6 Å^2^. Comparable ASADH-NADP binding was observed in *H. influenzae* (1PQU) and *V. cholerae* (3PZR) involving 13 hydrogen bonds, with buried surface areas of 505.8 Å^2^ and 502.8 Å^2^ respectively.

ASADH protein is previously described as having two states, apoenzyme and the NADP-inhibitor complex (with SMCS)^20^. Changes in enzyme conformation are attributed to NADP binding and involve the repositioning of two surface loops adjacent to the NADP binding site. Here, we show that the NADP binding of ASADH in P. aeruginosa shows a different orientation of the nicotinamide ring relative to other ASADH structures (Fig. 4C). The position of the nicotinamide ring is constrained through hydrogen bonds (Fig. 4A). As binding at the active site is predicted to cause a conformational change in the conserved loops enclosing the bound NADP, the difference in positioning likely affects the ability of NADP to stabilise the ASADH protein dimer. However, as the active site is relatively unchanged with NADP binding, active site binding is likely not significantly affected. This should be further explored using modelling approaches of docking inhibitors.

### Comparison to known structures

**Figure 5:**
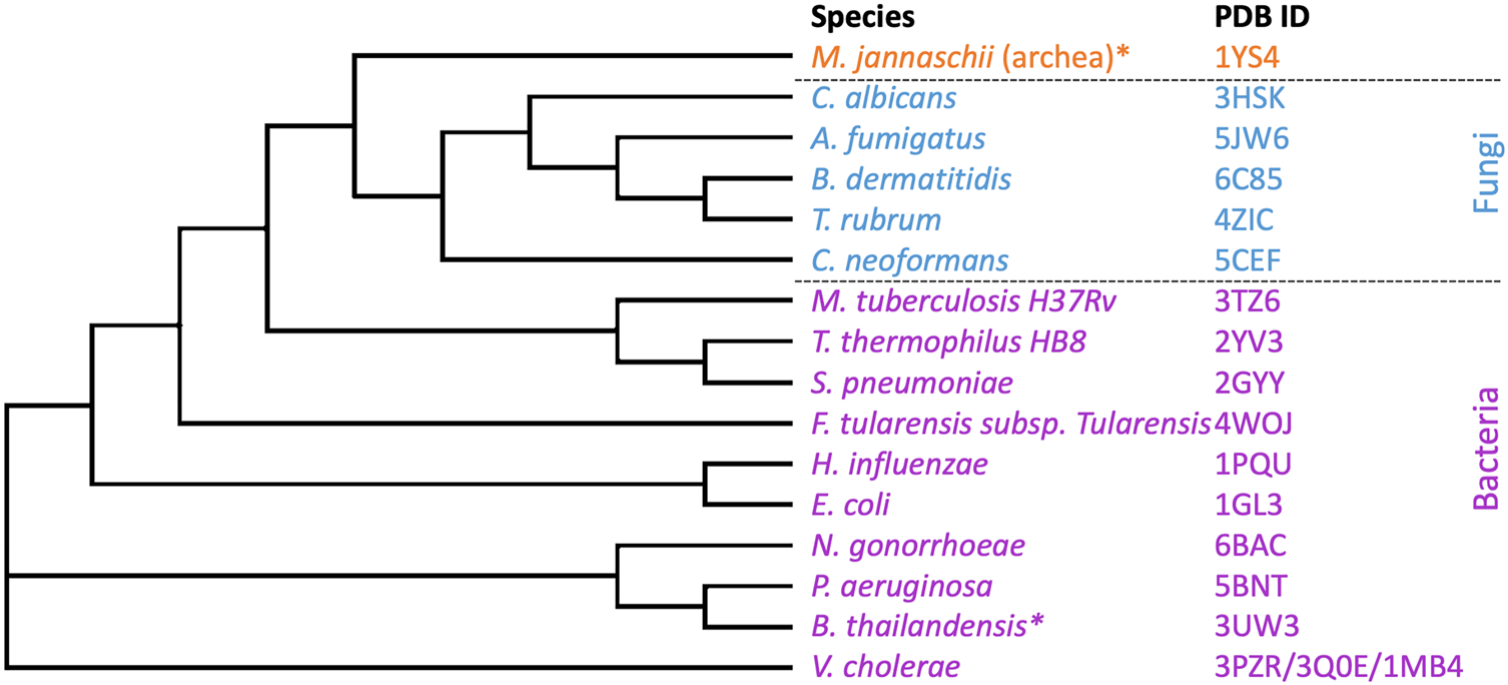
Phylogenic tree of ASADH proteins. The fungal (blue), bacterial (purple) and archaeal (orange) ASADH proteins and the corresponding PDB ID are shown. *An asterisk indicates a non-pathogenic species.

To determine the sequence similarity of the ASADH proteins to other ASADH proteins of known structures, Clustal Omega^49^ sequence alignment tools were used to generate a phylogenetic tree (Fig. 5). The selection of ASADH protein sequences for comparison was dependent on the availability of ASADH crystallized structures, which represent primarily organisms of pathogenic interest. *P. aeruginosa* and *N. gonorrhoeae* ASADH protein sequences are most closely related to ASADH proteins from *B. thailandensis* and *V. cholerae*.

To determine the most structural similar ASADH proteins available from those with structures available on the PDB, a homology search using DALI (heuristic PDB search) was performed^48^ using *N. gonorrhoeae* ASADH (6BAC) and *P. aeruginosa* (5BNT, chain B). Only one representative structure is listed for each organism (Table 2). The protein with the most sequence similarity to ASADH proteins from *P. aeruginosa* and *N. gonorrhoeae* is *B. thailandensis* ASADH (3UW3)^45^. This protein had a sequence similarity of 73% and RMSD of 0.8 Å and a sequence similarity of 71% and RMSD of 0.7 Å for *P. aeruginosa* and *N. gonorrhoeae* ASADH proteins respectively. This was expected for *P. aeruginosa* ASADH as *Burkholderia* (previously a part of *Pseudomonas*) is a gram-negative pathogenic group of bacteria that are closely related to *Pseudomonas*^50^*.* Notably, when comparing the two structures presented here, the NADP-bound ASADH from *P. aeruginosa* (5BNT) had an RMSD 0.8 Å and 68% sequence similarity to *N. gonorrhoeae* ASADH (6BAC).

The ASADH protein structures with the next most similar structural homology to the *P. aeruginosa* ASADH (5BNT) is *V. cholerae* ASADH*.* The *V. cholerae* ASADH protein was crystallized with NADP and product of S-carbamoyl-L-cysteine (3PZR), S-allyl-L-cysteine sulfoxide (3Q0E) and NADP and S-methyl-L-cysteine sulfoxide (1MB4)^6,20^. The crystal structures showed an RMSD 0.5 Å and sequence similarity 69% to *P. aeruginosa* ASADH (5BNT). Comparatively, ASADH protein from *N. gonorrhoeae* (6BAC) showed RMSD 0.8–0.9 Å and a sequence similarity of 66% to *V. cholerae* ASADH.

**Figure 6:**
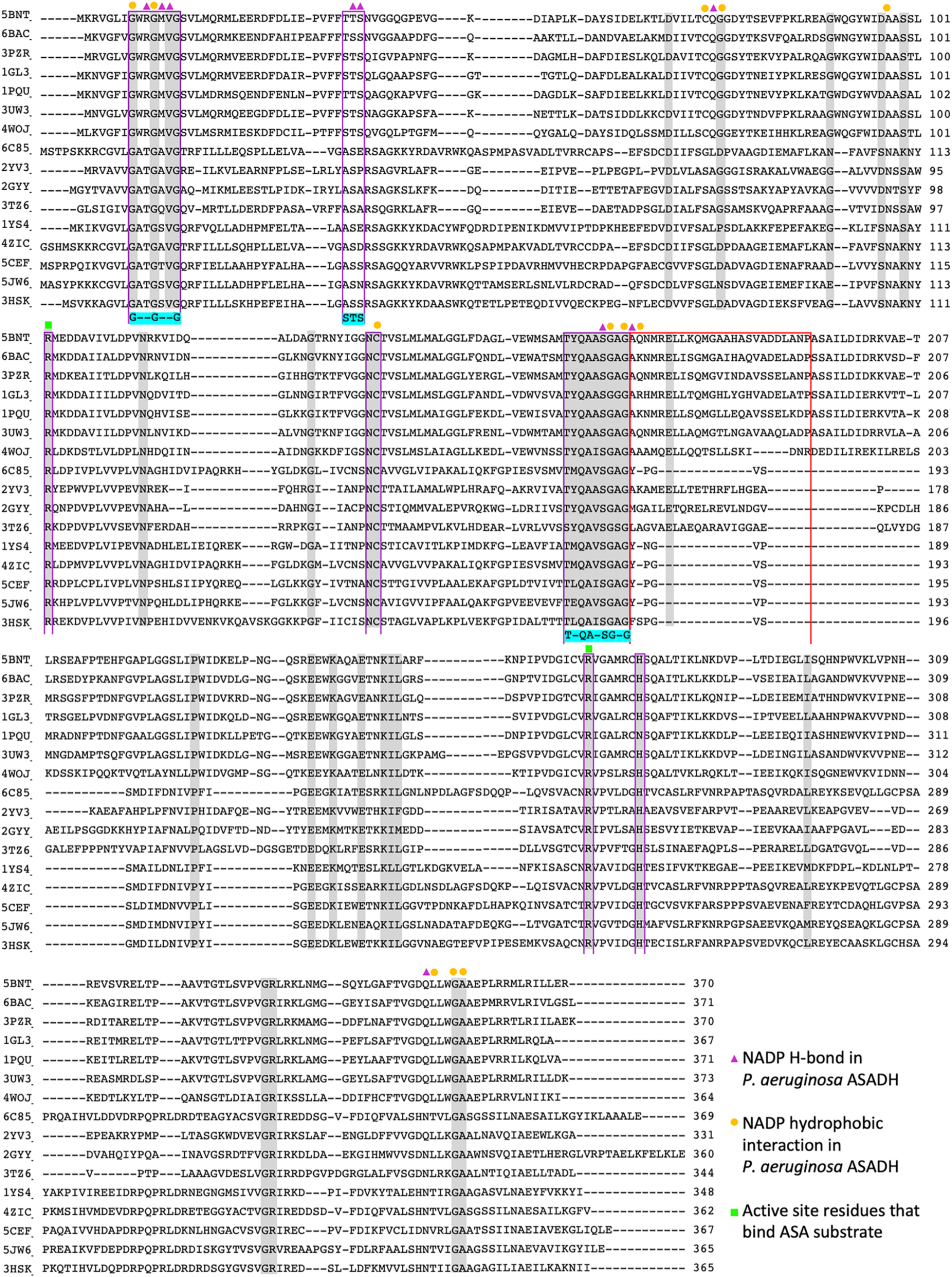
Sequence alignment of ASADH proteins. Highly conserved residues are highlighted in grey. The red box represents the helical subdomain region in the bacterial ASADH proteins. The conserved protein sequences for the STS, G–G–G and T-QA-SG-G binding motifs are highlighted in cyan. Protein sequences within the sequence alignment include *P. aeruginosa* (5BNT)*, N. gonorrhoeae* (6BAC)*, V. cholerae* (3PZR)*, E. coli* (1GL3)*, H. influenzae* (1PQU)*, B. thailandensis* (3UW3), *F. tularensis subsp. Tularen* (4WOJ)*, B. dermatitidis* (6C85)*, T. thermophilus HB8* (2YV3)*, S. pneumoniae* (2GYY)*, M. tuberculosis H37Rv* (3TZ6)*, M. jannaschii* (1YS4)*, T. rubrum* (4ZIC)*, C. neoformans* (5CEF)*, A. fumigatus* (5JW6) and *C. albicans* (3HSK).

A multiple sequence alignment was generated using the ASADH proteins identified as having a high structural similarity from the DALI heuristic PDB search, highlighting highly conserved residues and binding motifs of ASADH proteins (Fig. 6). Conserved protein sequences include a G–G–G binding motif for nucleoside binding and a T-QA-SG-G binding motif predicted to have a role in active site communication^5^. ASADH proteins also contain two conserved active site residues; Cys135, which initiates catalysis and His274 (H-bond interaction)^51^. The STS binding motif previously reported in NADP binding of ASADH proteins is not conserved in the *N. gonorrhoeae* ASADH (6BAC) sequence, which may affect binding affinity.

Studies to investigate the binding of inhibitors to ASADH both experimentally and using virtual modelling have predicted that a successful ASADH inhibitor will bind through electrostatic interactions with two highly conserved active site arginine residues^4^. Arginine residues within the active site from *S. pneumoniae* ASADH (Arg99, Arg245) and *V. cholerae* ASADH (Arg101, Arg267) bind the phosphate and the carboxyl group moieties of the aspartate β-semialdehyde (ASA) substrate, respectively. The residue Arg99-103 in bacterial ASADH proteins corresponds to Arg112-116 in fungal and archaeal ASADH proteins (Fig. 6, green box).

### SMCS binding to ASADH

**Figure 7:**
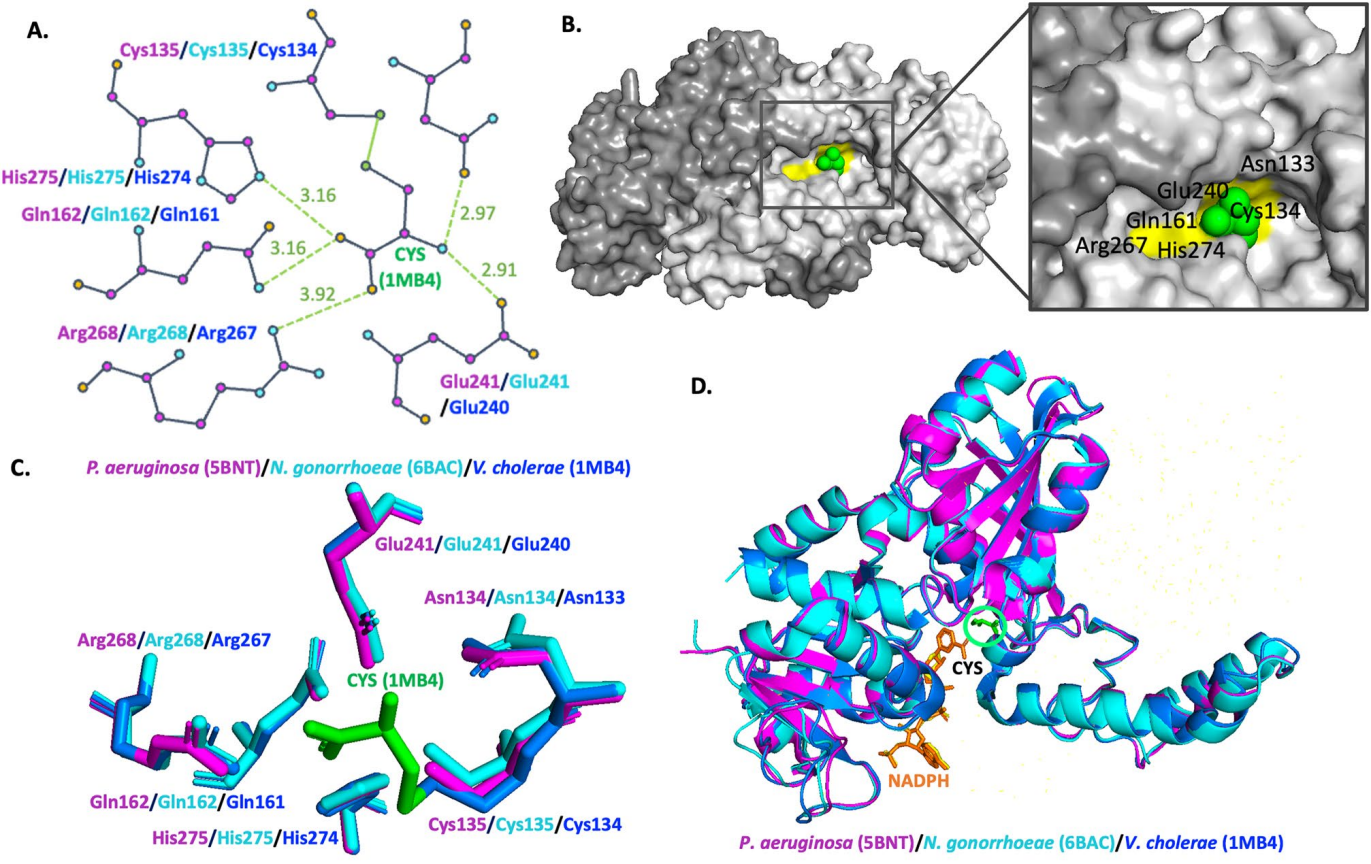
Comparison of active site inhibitor S-methyl-L-cysteine sulfoxide (SMCS) Cys binding in *V. cholerae* (1MB4), *P. aeruginosa* (5BNT) and *N. gonorrhoeae* (6BAC) ASADH proteins. (**A**) Schematic of H-bond interactions between active site residues of *V. cholerae* (1MB4, blue) ASADH and SMCS, conserved in *P. aeruginosa* (5BNT, purple) and *N. gonorrhoeae* (6BAC, cyan) ASADH proteins. (**B**) SMCS (Cys, green) bound within the *V. cholerae* (3PZR) ASADH dimer. Important active site residues for H-bonding within the NADP binding site cavity are shown (yellow). (**C**) Active site residues responsible for SMCS binding through H-bond interactions in *V. cholerae* ASADH (1MB4, blue). Cys (green) represents the covalently bound inhibitor, SMCS, in *V. cholerae* ASADH (1MB4) through the formation of a disulfide bond. Important active site residues are conserved in *P. aeruginosa* (5BNT, purple) and *N. gonorrhoeae* (6BAC, cyan) ASADH proteins. (**D**) Superimposed structures of *P. aeruginosa* (5BNT, purple), *N. gonorrhoeae* (6BAC, cyan) and *V. cholerae* (1MB4, blue).

To determine whether the binding pocket for an active site inhibitor of ASADH, S-methyl-L-cysteine sulfoxide (SMCS), in a structurally similar ASADH protein is conserved in *P. aeruginosa* (5BNT) and *N. gonorrhoeae* (6BAC) ASADH proteins, we performed a structural alignment of the binding pockets. Binding of SMCS was modelled from the *V. cholerae* ASADH (1MB4) structure (Fig. 7A,B). Inactivation of ASADH by SMCS (Cys) is a result of disulfide bond formation at Cys134. The H-bond interaction at His274 is also highly conserved and critical for mediating SMCS interaction. Other H-bond interactions with SMCS in *V. cholerae* ASADH (1MB4) also occur at Asn133, Gln161, Glu240 and Arg267 (Fig. 7C,D). The docking results show binding site similarity between existing literature and support the prediction that the SMCS molecule could be used to target the *P. aeruginosa* and *N. gonorrhoeae* ASADH proteins.

Several small molecule inhibitors of ASADH proteins with high binding efficiency have been identified (Table 3). Where some ASADH inhibitors are able to selectively inhibit ASADH in gram negative bacteria, other ASADH inhibitors have been shown to selectively inhibit fungal ASADH proteins^52^. There is an increasing need to design small molecule inhibitors that can selectively target pathogenic microorganisms that display antibiotic resistance.

**Table 3:** Ligands bound to ASADH proteins identified as structurally similar.

PDB ID	Organism	Ligands(s)
3PZR	*V. cholerae*	Product of S-carbamoyl-L-cysteine + NADP
3Q0E	*V. cholerae*	Product of S-allyl-L-cysteine sulfoxide
1MB4	*V. cholerae*	S-methyl-L-cysteine sulfoxide + NADPH
4R5M	*V. cholerae*	4-nitro-2-phosphono-benzoic acid + NADP
1GL3	*E. coli*	Substrate analogue s-methyl cysteine sulfoxide + NADPH
1PQU	*H. influenzae*	S-methyl cysteine sulfoxide + cacodylate + NADP
6C85	*B. dermatitidis*	p-benzoquinone
3TZ6	*M. tuberculosis H37Rv*	S-methyl-L-cysteine sulfoxide

*P. aeruginosa* has demonstrated antibiotic resistance to β-lactams, aminoglycosides, quinolones and other antibiotics^8^. Recently, whole genome sequencing of *Pseudomonas spp.* revealed antibiotic resistance genes to various antibiotics^28^. New antimicrobial drugs are required to treat *P. aeruginosa* infection and to combat antibiotic resistance^10^. New therapies that offer selective inhibition of key enzymes in pathogenic microorganisms may be an alternative or used in combination with current antibiotics^8^. Docking software can be used to predict structural and kinetic binding of small molecules*.* The ASADH protein from *V. cholerae* (1MB4) was used here to model SMCS binding within the newly solved structures of *P. aeruginosa* (5BNT) and *N. gonorrhoeae* (6BAC) ASADH proteins. This ASADH protein showed high structural and sequence similarity to the protein structures presented here, in particular, within conserved active site residues and binding site cavities. Both *P. aeruginosa* and *N. gonorrhoeae* ASADH proteins are potential targets for the small molecule inhibitor SMCS. Here, SMCS binding is modelled within ASADH, however, there are various other inhibitors, catalytic intermediates and substrate analogues including phosphonamidites analogues of the substrate aspartyl phosphate^53^ that could be modelled to predict binding. Selective inhibitors of ASADH proteins have been identified using screening tools and a fragment library containing 378 compounds^52,54,55^, virtual screening of databases^23^ and molecular docking approaches^4,56^. Due to structural similarities with other characterised ASADH proteins, we predict that various other ligands could be modelled within ASADH protein structures presented in this paper via screening of inhibitors shown to bind ASADH proteins.

## Conclusion

Antimicrobial drug resistance is an urgent and ongoing medical threat. High-resolution structures of enzymes that are critical for microbial survival represent an important starting point for the development of new antibiotics. Here we present the crystallographic structures *P. aeruginosa* and *N. gonorrhoeae* ASADH proteins, that represent ideal platforms for potential in silico drug screening.

## References

[1] Viola RE (2001). The central enzymes of the aspartate family of amino acid biosynthesis. Acc. Chem. Res..

[2] Dahal GP, Viola RE (2017). Structure of a fungal form of aspartate-semialdehyde dehydrogenase from *Aspergillus fumigatus*. Acta Crystallogr. F Struct. Biol. Commun..

[3] Kumar R, Garg P, Bharatam PV (2016). Pharmacoinformatics analysis to identify inhibitors of Mtb-ASADH. J. Biomol. Struct. Dyn..

[4] Luniwal A, Wang L, Pavlovsky A, Erhardt PW, Viola RE (2012). Molecular docking and enzymatic evaluation to identify selective inhibitors of aspartate semialdehyde dehydrogenase. Bioorg. Med. Chem..

[5] Mank NJ (2018). Structure of aspartate β-semialdehyde dehydrogenase from *Francisella tularensis*. Acta Crystallogr. F Struct. Biol. Commun..

[6] Pavlovsky AG, Liu X, Faehnle CR, Potente N, Viola RE (2012). Structural characterization of inhibitors with selectivity against members of a homologous enzyme family. Chem. Biol. Drug Des..

[7] Kobayashi K (2003). Essential *Bacillus subtilis* genes. Proc. Natl. Acad. Sci. USA.

[8] Pang Z, Raudonis R, Glick BR, Lin TJ, Cheng Z (2019). Antibiotic resistance in *Pseudomonas aeruginosa*: Mechanisms and alternative therapeutic strategies. Biotechnol. Adv..

[9] Foca M (2000). Endemic *Pseudomonas aeruginosa* Infection in a Neonatal Intensive Care Unit. N. Engl. J. Med..

[10] Pachori P, Gothalwal R, Gandhi P (2019). Emergence of antibiotic resistance *Pseudomonas aeruginosa* in intensive care unit; a critical review. Genes Dis..

[11] Nguyen, H. V. N., Smith, M.E., & Hayoun, M.A. *Glanders and Melioidosis*, (2020). 28846298

[12] Ng LK, Martin IE (2005). The laboratory diagnosis of *Neisseria gonorrhoeae*. Can. J. Infect. Dis. Med. Microbiol..

[13] Bala M, Ray K, Gupta SM, Muralidhar S, Jain RK (2007). Changing trends of antimicrobial susceptibility patterns of *Neisseria gonorrhoeae* in India and the emergence of ceftriaxone less susceptible *N. gonorrhoeae* strains. J. Antimicrob. Chemother..

[14] Bala M, Sood S (2010). Cephalosporin resistance in *Neisseria gonorrhoeae*. J. Glob. Infect. Dis..

[15] Tanaka M (2006). Analysis of mutations within multiple genes associated with resistance in a clinical isolate of *Neisseria gonorrhoeae* with reduced ceftriaxone susceptibility that shows a multidrug-resistant phenotype. Int. J. Antimicrob. Agents.

[16] Tapsall JW, Ndowa F, Lewis DA, Unemo M (2009). Meeting the public health challenge of multidrug- and extensively drug-resistant *Neisseria gonorrhoeae*. Expert Rev. AntiInfect. Ther..

[17] Unemo M, Del Rio C, Shafer WM (2016). Antimicrobial resistance expressed by *Neisseria gonorrhoeae*: A major global public health problem in the 21st century. Microbiol. Spectr..

[18] Jarvis GA, Chang TL (2012). Modulation of HIV transmission by Neisseria gonorrhoeae: Molecular and immunological aspects. Curr. HIV Res..

[19] Organization, W. H. *Global Action Plan on Antimicrobial Resistance*, (2015).

[20] Blanco J, Moore RA, Kabaleeswaran V, Viola RE (2003). A structural basis for the mechanism of aspartate-beta-semialdehyde dehydrogenase from *Vibrio cholerae*. Prot. Sci..

[21] Hadfield A (1999). Structure of aspartate-beta-semialdehyde dehydrogenase from *Escherichia coli*, a key enzyme in the aspartate family of amino acid biosynthesis. J. Mol. Biol..

[22] Faehnle CR, Le Coq J, Liu X, Viola RE (2006). Examination of key intermediates in the catalytic cycle of aspartate-beta-semialdehyde dehydrogenase from a gram-positive infectious bacteria. J. Biol. Chem..

[23] Kumar R, Garg P, Bharatam PV (2015). Shape-based virtual screening, docking, and molecular dynamics simulations to identify Mtb-ASADH inhibitors. J. Biomol. Struct. Dyn..

[24] Faehnle CR, Ohren JF, Viola RE (2005). A new branch in the family: structure of aspartate-beta-semialdehyde dehydrogenase from *Methanococcus jannaschii*. J .Mol. Biol..

[25] Arachea BT, Liu X, Pavlovsky AG, Viola RE (2010). Expansion of the aspartate beta-semialdehyde dehydrogenase family: The first structure of a fungal ortholog. Acta Crystallogr. D Biol. Crystallogr..

[26] Dahal G, Viola RE (2015). Structure of a fungal form of aspartate semialdehyde dehydrogenase from *Cryptococcus neoformans*. Acta Crystallogr. F Struct. Biol. Commun..

[27] Viola RE, Liu X, Ohren JF, Faehnle CR (2008). The structure of a redundant enzyme: A second isoform of aspartate beta-semialdehyde dehydrogenase in *Vibrio cholerae*. Acta Crystallogr. D Biol. Crystallogr..

[28] Meng L (2020). Antibiotic resistance patterns of *Pseudomonas* spp. isolated from raw milk revealed by whole genome sequencing. Front. Microbiol..

[29] Galán JE, Nakayama K, Curtiss R (1990). Cloning and characterization of the asd gene of *Salmonella typhimurium:* Use in stable maintenance of recombinant plasmids in Salmonella vaccine strains. Gene.

[30] Akerley BJ (2002). A genome-scale analysis for identification of genes required for growth or survival of *Haemophilus influenzae*. Proc. Natl. Acad. Sci. USA.

[31] Salama NR, Shepherd B, Falkow S (2004). Global transposon mutagenesis and essential gene analysis of *Helicobacter pylori*. J. Bacteriol..

[32] Gerdes SY (2003). Experimental determination and system level analysis of essential genes in *Escherichia coli* MG1655. J. Bacteriol..

[33] Becker D (2006). Robust Salmonella metabolism limits possibilities for new antimicrobials. Nature.

[34] Breijyeh Z, Jubeh B, Karaman R (2020). Resistance of gram-negative bacteria to current antibacterial agents and approaches to resolve it. Molecules.

[35] González-Bello C (2017). Antibiotic adjuvants: A strategy to unlock bacterial resistance to antibiotics. Bioorg. Med. Chem. Lett..

[36] Laws M, Shaaban A, Rahman KM (2019). Antibiotic resistance breakers: Current approaches and future directions. FEMS Microbiol. Rev..

[37] Bryan CM (2011). High-throughput protein production and purification at the Seattle structural genomics center for infectious disease. Acta Crystallogr. Sect. F Struct. Biol. Cryst. Commun..

[38] Kabsch W (2010). XDS. Acta Crystallogr. Sect. D.

[39] Kabsch W (2010). Integration, scaling, space-group assignment and post-refinement. Acta Crystallogr. Sect. D.

[40] Long F, Vagin AA, Young P, Murshudov GN (2008). BALBES: a molecular-replacement pipeline. Acta. Crystallogr. D Biol. Crystallogr..

[41] Adams PD (2010). PHENIX: A comprehensive Python-based system for macromolecular structure solution. Acta Crystallogr. Sect. D Biol. Crystallogr..

[42] SeeSAR (Sankt Augustin, Germany, 2021).

[43] Holm L, Laakso LM (2016). Dali server update. Nucleic Acids Res..

[44] Hanukoglu I (2015). Proteopedia: Rossmann fold: A beta-alpha-beta fold at dinucleotide binding sites. Biochem. Mol. Biol. Educ..

[45] Baugh L (2013). Combining functional and structural genomics to sample the essential Burkholderia structome. PLoS ONE.

[46] Krissinel E, Henrick K (2007). Inference of macromolecular assemblies from crystalline state. J. Mol. Biol..

[47] Laskowski RA (2009). PDBsum new things. Nucleic Acids Res..

[48] Holm L (2019). Benchmarking fold detection by DaliLite v.5. Bioinformatics.

[49] Sievers F (2011). Fast, scalable generation of high-quality protein multiple sequence alignments using Clustal Omega. Mol. Syst. Biol..

[50] Yabuuchi E (1992). Proposal of Burkholderia gen. nov. and transfer of seven species of the genus Pseudomonas homology group II to the new genus, with the type species *Burkholderia cepacia* (Palleroni and Holmes 1981) comb. nov. Microbiol. Immunol..

[51] Blanco J, Moore RA, Faehnle CR, Viola RE (2004). Critical catalytic functional groups in the mechanism of aspartate-[beta]-semialdehyde dehydrogenase. Acta Crystallogr. Sect. D.

[52] Gao G, Liu X, Pavlovsky A, Viola RE (2010). Identification of selective enzyme inhibitors by fragment library screening. J. Biomol. Screen..

[53] Cox RJ, Gibson JS, MayoMartín MB (2002). Aspartyl phosphonates and phosphoramidates: the first synthetic inhibitors of bacterial aspartate-semialdehyde dehydrogenase. ChemBioChem.

[54] Sarver JG (2012). Early stage efficacy and toxicology screening for antibiotics and enzyme inhibitors. J. Biomol. Screen..

[55] Thangavelu B, Bhansali P, Viola RE (2015). Elaboration of a fragment library hit produces potent and selective aspartate semialdehyde dehydrogenase inhibitors. Bioorg. Med. Chem..

[56] Wang X (2021). IMB-XMA0038, a new inhibitor targeting aspartate-semialdehyde dehydrogenase of Mycobacterium tuberculosis. Emerg. Microbes Infect..

